# Reframing Solitude as a Strategy to Reduce Loneliness

**DOI:** 10.1093/geroni/igaf122.369

**Published:** 2025-12-31

**Authors:** Jana Nikitin

**Affiliations:** University of Vienna, Vienna, Wien, Austria

## Abstract

Loneliness is a significant societal concern with profound effects on physical and mental health. Older adults can be particularly vulnerable due to life transitions, reduced social networks, and increased time spent alone. However, while solitude may become more prevalent with age, it does not inherently lead to loneliness. Learning to experience solitude positively could offer a meaningful way to enhance well-being. This study tested a brief strategy designed to reframe solitude as a positive experience, leveraging the saying-is-believing effect to reduce loneliness. Using an experimental design in an online study (N = 211 participants aged 18–89), participants were randomly assigned to one of two groups. The experimental group actively reflected on the positive opportunities solitude offers for pursuing intrinsically motivated goals, while the control group simply described their emotions during solitude. Findings revealed significantly lower levels of loneliness in the experimental group compared to the control group. Mediation analysis identified self-efficacy as a key mechanism driving this effect. Importantly, these results do not suggest that social isolation is merely an individual responsibility; rather, they highlight the potential of psychological reframing as a complementary approach in loneliness prevention. By shifting perspectives on solitude, this research provides valuable insights into fostering resilience in older adults and underscores the need for scalable, cost-effective solutions that promote well-being without overlooking the structural factors contributing to social isolation.

